# Examining variations in hospital productivity in the English NHS

**DOI:** 10.1007/s10198-014-0569-5

**Published:** 2014-02-25

**Authors:** Adriana Castelli, Andrew Street, Rossella Verzulli, Padraic Ward

**Affiliations:** 1 Centre for Health Economics, University of York, Alcuin A Block, York, YO10 5DD UK; 2 Scuola Superiore di Politiche per la Salute, Università di Bologna, Bologna, Italy; 3 Irish Centre for Social Gerontology, National University of Ireland, Galway, Galway, Ireland

**Keywords:** Hospital sector, Productivity, Production functions, DEA, C21, C43, D24, I11, I18

## Abstract

**Objectives:**

Numerous papers have measured hospital efficiency, mainly using a technique known as data envelopment analysis (DEA). A shortcoming of this technique is that the number of outputs for each hospital generally outstrips the number of hospitals. In this paper, we propose an alternative approach, involving the use of explicit weights to combine diverse outputs into a single index, thereby avoiding the need for DEA.

**Methods:**

Hospital productivity is measured as the ratio of outputs to inputs. Outputs capture quantity and quality of care for hospital patients; inputs include staff, equipment, and capital resources applied to patient care. Ordinary least squares regression is used to analyse why output and productivity varies between hospitals. We assess whether results are sensitive to consideration of quality.

**Results:**

Hospital productivity varies substantially across hospitals but is highly correlated year on year. Allowing for quality has little impact on relative productivity. We find that productivity is lower in hospitals with greater financial autonomy, and where a large proportion of income derives from education, research and development, and training activities. Hospitals treating greater proportions of children or elderly patients also tend to be less productive.

**Conclusions:**

We have set out a means of assessing hospital productivity that captures their multiple outputs and inputs. We find substantial variation in productivity among English hospitals, suggesting scope for productivity improvement.

**Electronic supplementary material:**

The online version of this article (doi:10.1007/s10198-014-0569-5) contains supplementary material, which is available to authorized users.

## Introduction

Numerous articles have appeared over the years purporting to measure differences in hospital efficiency, the majority of which apply a technique known as data envelopment analysis (DEA) [1]. Few have had any practical influence, either on policy makers or hospital management [2, 3]. This lack of impact stems largely from concerns about the robustness of the technique and the limited insights it offers about what action to take [4].

Data envelopment analysis has proved popular among academics because it can accommodate analysis of multiple outputs and inputs. It does this by applying linear programming to search for a set of organisation-specific weights with which to combine diverse outputs into a single function (and the same for inputs). However, DEA can only handle multiple outputs up to a point determined by the number of organisations under consideration [5]. As Newhouse noted, the problem with applying this technique to the hospital sector is that the number of outputs produced by each hospital usually outstrips the number of hospitals under consideration [6]. Newhouse had in mind each output being described as a diagnosis related group (DRG), of which there were some 500 at that time of writing. There are few countries with this many hospitals. One “solution” by proponents of DEA has been to describe hospital outputs much more crudely, as the numbers of inpatients, day-cases or outpatients [7–9]. This fails to recognise the substantial heterogeneity among patients within these categories, thereby immediately undermining the exercise: results may simply reflect analytical failure to take proper account of the true nature of production.

In this paper we propose an alternative approach to dealing with the multiple output problem. This involves imposing an explicit set of weights with which to combine diverse outputs into a single index, thereby obviating the need for DEA. Our approach is an extension of the national accounting framework, developed to measure changes in health care productivity at national level. Our interest here is in measuring relative productivity among hospitals, and we develop cross-sectionally equivalent specifications of national output and input indices. We set out these specifications in the “Methods” section. We then apply these methods to the English hospital sector, describing our data in the “Data” section. Results are reported in the “Results” section. The last section concludes.

## Methods

In this paper we pursue two main objectives. First, we construct measures of productivity for each NHS hospital. To this end we follow the approach adopted in the construction of the national productivity index detailed in Dawson et al. [10] and Castelli et al. [11]. We construct these measures for the financial years 2008/09 and 2009/10. Second, we analyse why productivity varies from one hospital to another by specifying econometric models in which productivity is regressed against a variety of variables that capture characteristics of each hospital. As sensitivity analyses, we estimate Cobb–Douglas production functions with output as the dependent variable and we assess the impact of accounting for quality when measuring hospital output.

### Hospital productivity

Productivity is measured by comparing the total amount of health care ‘output’ produced to the total amount of ‘input’ used to produce this output (see Eq. 1). Output consists of all healthcare provided to patients (both in inpatient and outpatient settings) by hospital *h* (*h* = 1…*H)* and inputs include the staff, intermediate, and capital resources that contribute to the production of healthcare for these patients.

1 $${\text{Productivity of hospital }}h\, = \frac{{  {\text{Outputs}}_{h} }}{{{\text{Inputs}}_{h} }}$$

To ease interpretation and comparison of productivity across hospitals, for each year we construct a measure of standardised productivity (*P*_*h*_) for each hospital *h*, defined as^1^:

2 $$P_{h} = \left\{ {\left[ {\left( {\frac{{X_{h} }}{{Z_{h} }}} \right)/\frac{1}{H}\mathop \sum \limits_{h} \frac{{X_{h} }}{{Z_{h} }}} \right] - 1} \right\} \times 100,$$

where *X*_*h*_ is the volume of output produced and *Z*_*h*_ the amount of input used in hospital *h*. The standardised productivity of each hospital is given by dividing the hospital specific output/input ratio by the national average output/input ratio, standardising around 1 and expressing this as a percentage difference. Thus, if standardised productivity in hospital *h* is 10, this means that productivity in that hospital is 10 % higher than the national average.

### Measuring hospital outputs

Hospital output consists primarily of the number of patients treated. Patients have diverse health care needs and the nature of the care received differs markedly from one patient to the next. We take this diversity into account by classifying inpatients into one of 1,400 healthcare resource groups (HRGs), the English equivalent of DRGs, and outpatient attendances into 1,498 categories [12].

Healthcare resource groups and the outpatient groups form the building blocks of activity-based funding in England by which hospitals are paid a prospective price for each patient treated in each output category [13]. The HRG prices are based on the national average cost reported 3 years previously for all patients categorised to the HRG in question [14]. Consistent with this payment policy, we use national average costs as a set of weights to distinguish patients categorised to different HRGs and outpatient groups and to aggregate the total number of patients treated by each hospital into an overall measure of hospital output. Thus, ‘cost-weighted’ hospital output *X*_*h*_^*c*^ is defined as:

3 $$X_{h}^{c} = \mathop \sum \limits_{j = 1}^{J} x_{jh} \bar{c}_{j} ,$$

where *x*_*jh*_ represents the number of patients categorised to output category *j* with *j* = 1…*J* in hospital *h*. The cost weight is defined as $\bar{c}_{j} = c_{j} /\hat{c}$ where *c*_*j*_ represents the national average cost for patients allocated to output *j* and $\hat{c}$ is the national average cost across all patients.

Of course, it is not enough that hospitals treat patients, they should also treat them well. However, evidence suggests considerable variability across hospitals in the quality of care that patients experience [15–18]. The quality of treatment can be recognised in the measure of output, such that a hospital that delivers superior quality to its patients is deemed to have produced a greater amount of output. A simple way to do this is to introduce quality as a scalar to cost-weighted output:

4 $$X_{h} = \mathop \sum \limits_{j = 1}^{J} x_{jh} \bar{c}_{j} \bar{q}_{jh} ,$$

where the quality adjustment is identified by the term $\bar{q}_{jh} = q_{jh} /\hat{q}_{j}$. Here $q_{jh}$ is the quality of care experienced by patients allocated to output *j* in hospital *h* and $\hat{q}_{j}$ captures the national average quality of output *j*. Our adjustment is designed to reflect the quality-adjusted life years (QALYs) associated with treatment and adapts the form used to account for quality in the English national accounts [10]:

5 $$\bar{q}_{jh} = \left( {\frac{{a_{jh} - k_{j} }}{{\widehat{{a_{jh} - k_{j} }}}}} \right)\frac{{\left[ {\frac{{\left( {1 - e^{{ - r_{Q} LE_{jh} }} } \right)}}{{r_{Q} }} - \frac{{\left( {e^{{r_{W} W_{jh} }} - 1} \right)}}{{r_{W} }}} \right]}}{{\left[ {\frac{{\left( {1 - e^{{ - r_{Q} \widehat{LE}_{j} }} } \right)}}{{r_{Q} }} - \frac{{\left( {e^{{r_{W} \hat{W}_{j} }} - 1} \right)}}{{r_{W} }}} \right]}} .$$

Direct QALY estimates for each HRG are unavailable. Instead, we construct the equivalent of a QALY profile for patients allocated to each HRG [19]. A survival measure (*a*_*jh*_) captures the probability of survival for people in each HRG. We multiply this probability by life expectancy (*LE*_*jh*_) and a measure of change in health status following treatment (*k*_*j*_) to arrive at an estimate of the total amount of QALYs experienced by this group of survivors over their remaining lifetime. Those who do not survive treatment are afforded a zero QALY gain. Waiting for treatment (*w*_*jh*_) yields disutility, and we express this disutility in terms of QALYs by valuing days spent waiting in the same metric as we value remaining life expectancy. This allows us to subtract the disutility associated with waiting from the QALY gains associated with treatment in order to arrive at our estimate of net QALY gain for each HRG.

Survival (*a*_*jh*_) is measured as the 30-day post discharge survival rates for each output in each hospital. The change in health status (*k*_*j*_) is measured as the ratio of average health status (*h* ^0^) before and after (h*) treatment, such that $$k_{j} = {\raise0.7ex\hbox{${h_{j}^{0} }$} \!\mathord{\left/ {\vphantom {{h_{j}^{0} } {h_{j}^{*} }}}\right.\kern-0pt} \!\lower0.7ex\hbox{${h_{j}^{*} }$}}$$. In the absence of HRG-specific information we assume that, on average, the ratio for elective patients is twice that for non-elective patients [20]. Life expectancy (*LE*_*jh*_) associated with each HRG is calculated by considering the age and gender profiles of patients allocated to each HRG. The inverse exponential function reflects decreasing life expectancy over time and *r*_*Q*_ is the discount rate applied to future life years.

Waiting times (*w*_*jh*_) for each HRG in each hospital are measured at the 80th percentile of the distribution for patients categorised to each HRG. Our formulation implies that delays to treatment have adverse health consequences and that the marginal disutility of waiting increases as the delay increases, with the disutility captured as an exponential function and by the discount rate *r*_*w*_ [10].

### Measuring hospital inputs

The provision of hospital treatment involves utilising a variety of different inputs during the production process. These inputs include labour, capital and intermediate inputs. Capital is defined as any non-labour input with an asset life of more than a year, such as land and buildings. Intermediate inputs comprise all other non-labour inputs, such as drugs and dressings, disposable supplies and equipment, and use of utilities.

Information about the physical quantities of these inputs is hard to come by, but comprehensive details are available about how much hospitals spend on each type of input. Total expenditure can be broken down as follows:

6 $$Z_{h}^{'} = E_{h}^{L} + E_{h}^{A} + E_{h}^{K} + E_{h}^{M} ,$$

where *Z*_*h*_^′^ is an aggregation of expenditure on NHS labour (*E*_*h*_^*L*^), agency staff (*E*_*h*_^*A*^), capital (*E*_*h*_^*K*^) and intermediate inputs (*E*_*h*_^*M*^).

Hospital expenditure is the product of the volume and price of its inputs. Prices of labour, buildings and land may vary across English hospitals according to their geographical location. In order to remove these exogenous price effects, we apply the sub-indices of the Department of Health’s Market Forces Factor (MFF) to expenditure on labour (*θ*_*h*_^*L*^) and capital (*θ*_*h*_^*K*^) inputs [21]. Intermediate inputs are not considered to be subject to similar exogenous geographical influences and hence no adjustment is made for them.

Our measure of total hospital input, then, is calculated as:

7 $$Z_{h}^{{}} = \theta_{h}^{L} (E_{h}^{L} + E_{h}^{A} ) + \theta_{h}^{K} E_{h}^{K} + E_{h}^{M}$$

### Productivity indices

In summary, we construct two standardised productivity measures for each hospital. Our preferred measure of total factor productivity, set out as Eq. (2), uses Eq. (4) to construct the output index and Eq. (7) for the input index. We also construct a productivity measure which does not account for quality. This involves replacing the output index given by Eq. (4) with that of Eq. (3). The productivity measure becomes:

8 $$P_{h}^{c} = \left\{ {\left[ {\left( {\frac{{X_{h}^{c} }}{{Z_{h} }}} \right)/\frac{1}{H}\mathop \sum \limits_{h} \frac{{X_{h}^{c} }}{{Z_{h} }}} \right] - 1} \right\} \times 100$$

### Examining variations in hospital productivity

We examine variations in hospital productivity by estimating ordinary least squares (OLSs) regressions with robust standard errors to account for potential heteroscedasticity. Our dependent variables are the two standardised productivity measures described in Eqs. (2) and (8) i.e. *y*_*h*_ = {*P*_*h*_, *P*_*h*_^*C*^}. We regress these against a number of explanatory variables ($g = 1, \ldots , G$ *)* that have been identified in the literature as exerting an influence over performance at hospital level. The OLS regression model is given by:

9 $$y_{h} = \beta_{0} + \mathop \sum \limits_{g = 1}^{10} \beta_{g} Hospvars_{gh} + \varepsilon_{h}.$$

We test the relationship between productivity and the proportion of each hospital’s patients that received some form of specialised care (*Spec*). These patients were identified using the approach described in Daidone and Street [22]. The effect of specialisation on hospital productivity is hard to determine. In theory, hospitals that offer a wide range of hospital services might benefit from economies of scope, in that the joint production of outputs yields cost savings [23]. However, specialist hospitals might be more productive because resources are ear-marked for specific functions rather than being subject to competing use and because specialisation promotes the development of expertise (Harris [24], Kjekshus and Hagen [25] and Street et al. [26, 27]).

Public NHS hospitals can be divided into Foundation Trusts (FTs) and non-Foundation Trusts (NFTs). FTs were introduced in the English NHS in 2004/05, as not-for-profit public organisations which enjoy a greater managerial and financial autonomy from direct central government control [28]. FTs can retain surpluses (to re-invest in capital equipment and/or to increase salaries) and can borrow money to invest in improved services for patients and service users [29]. Moreover, FTs have a new form of governance designed to create a greater engagement of the local community, patients and staff in running their activities. The expectation is that these incentives would allow FTs to deliver “high productivity, greater innovation and greater job satisfaction” [30, 31].

Teaching hospitals might incur higher costs and appear less productive than non-teaching hospitals because they tend to treat more complex or more severe patients. Moreover, teaching might introduce delays to the treatment process, as consultants tend to spend more time when assessing a patient in order to train medical students [32]. In many studies, hospitals are classified simply as teaching hospitals or not. Here, rather than using a dummy for teaching status, we identify teaching activities as a continuous variable, measuring income received by hospitals for education, research and development, and training as a proportion of total income (*Education_p*).

Hospitals that care for a large proportion of patients admitted as emergencies may find it more challenging to optimise utilisation of their facilities [33, 34]. Hence, we control for the proportion of emergency admissions over total admissions (*Emerg_p*).

Healthcare resource groups (HRGs) do not capture perfectly differences in care requirements among patients. Recognising this, we consider some variables capturing patient case-mix. These include the percentage of female patients (*Female_p*) and the percentage of patients falling into three age categories: aged 0–15 years (*Age_015_p*), aged 46–60 years (*Age_4660_p*) and over 60 years (*Age_60_p*), with patients aged 16–45 years (*Age_1645_p*) forming the reference category.

We consider two variables that capture efficiency in resource use. These are the proportion of occupied beds over total beds (*Occuppc*) and the average length of stay, which is calculated as the ratio of total inpatient days over total number of patients (LoS).

### Examining variations in hospital outputs

Hospital productivity specified as a ratio imposes an implicit assumption of constant returns to scale. This assumption may not hold so, as a sensitivity analysis, we estimate a standard Cobb–Douglas production function to examine variations in the log of hospital output (both cost and quality adjusted).

With three factors of production a Cobb–Douglas production function can be specified as:

10 $${ \ln }Y_{h} = { \ln }S_{h} + \gamma_{1} { \ln }\left[ {\theta_{h}^{L} \left( {E_{h}^{L} + E_{h}^{A} } \right)} \right] + \gamma_{2} { \ln }\left( {\theta_{h}^{K} E_{h}^{K} } \right) + \gamma_{3} { \ln }E_{h}^{M} + \varepsilon_{h} ,$$

where $\gamma_{1} , \gamma_{2}$ and $\gamma_{3}$ are parameters describing the contributions to output made by labour, capital and intermediate inputs, respectively. It is assumed that the parameters $\gamma_{1} , \gamma_{2}$ and $\gamma_{3}$ are the same for all hospitals, with differences amongst hospitals being captured by the error term *ɛ*_*h*_. The logarithmic form enables us to interpret coefficients as elasticities: for example, a 1 % increase in the amount of total labour employed is predicted to lead to a percentage increase in output equal to the value *γ*_1_. Further, *S*_*h*_ can be thought of in terms of a “shift” parameter comprising the explanatory variables discussed in the section “Examining variations in hospital productivity”.

We estimate two separate equations. In the first, we assume that only the three factors of production influence hospital outputs; in the second, we also include the control variables (*hospvars*) from the section “Examining variations in hospital productivity”.

## Data

We construct a range of variables about (1) hospital inpatient activity using data extracted from the hospital episode statistics (HES) database [35] and (2) outpatient attendances from the reference cost database [36, 37]. The HES database comprises more than 15 million patient records per financial year, with each record reported as a finished consultant episode (FCEs). An FCE measures the time a patient spends under the care of a particular consultant. The majority (around 88 %) of patients remain under the care of the same consultant for the whole duration of their hospital stay; however, a small proportion is cared for by more than one consultant because they are transferred from one specialty to another. By combining the episodes of care received by each individual patient, we construct a “provider spell” for each patient, capturing their entire hospital stay.

To construct our measures of quality, we merge date of death data collated by the Office of National Statistics and life expectancy tables [38] to patients in the HES database. This allows us to capture deaths occurring within 30 days from discharge and to construct age and gender-specific measures of life expectancy. Waiting times and length of stay are calculated directly from HES. Each FCE is associated with an HRG; we allocate patients with multiple episodes to the HRG recorded in their first FCE.

We assign a cost to each FCE in HES and to each outpatient attendance using the national average unit costs reported in the reference cost data. The cost of a spell is calculated on the basis of the most expensive FCE within the spell [11]. We then calculate the national average cost of a patient spell for each HRG. These national averages form the set of cost weights *c*_*j*_ by which to aggregate patients in different HRGs and outpatient categories into a single index of output.

Information about the inputs used in the production of hospital activity is taken from the hospital financial accounts. These detail expenditure on NHS and agency staff by broad categories of labour input, such as medical and nursing staff, technical and clerical staff, and managers. Intermediate inputs include drugs and gases, clinical supplies, catering, hotel services, laundry, bedding, energy, establishment and premises costs. Two forms of information are reported about capital expenditure: current outlays on equipment and depreciation on assets. We make assumptions according to the asset in question about what proportion of current expenditure is employed in the current period [39]. As mentioned, we adjust reported costs for labour, buildings and land using the MFF.

Tables 1 and 2 provide summary statistics about hospital inpatient and outpatient activity and about inputs for the years 2008/09 and 2009/10, respectively. Note that five of the hospitals that appear in 2008/09 were merged into two hospitals in 2009/10.^2^ Table 3 reports descriptive statistics of each explanatory variable used in the regression analysis, including its source.^3^

**Table 1:** Summary statistics for NHS outputs and inputs, 2008/09

Variable	Obs	Mean	SD	Min	Max
*Hospital outputs*					
Elective and day cases					
Number of patients	169	48,326	34,218	3,416	200,977
Mean 30-day post discharge survival rate	169	0.99	0.00	0.97	1.00
Mean life expectancy in years	169	24	7	15	63
80th percentile waiting times (days)	168	72	63	16	750
Non-electives					
Number of patients	169	41,135	22,921	203	127,522
Mean 30-day post discharge survival rate	169	0.95	0.02	0.83	1.00
Mean life expectancy in years	169	34	7	17	65
Outpatient					
Volume of activity	169	388,465	208,270	31,075	1,044,235
*Hospital inputs (£000)*					
NHS labour	169	150,652	92,185	10,184	548,360
Agency labour	169	6,171	5,882	0	44,887
Intermediate goods and services	169	55,899	42,887	6,553	234,753
Capital	169	19,028	15,130	269	115,739

**Table 2:** Summary statistics for NHS outputs and inputs, 2009/10

Variable	Obs	Mean	SD	Min	Max
*Hospital outputs*					
Elective and day cases					
Number of Patients	166	49,183	34,235	3,344	200,917
Mean 30-day post discharge survival rate	166	0.99	0.00	0.97	1.00
Mean life expectancy in years	166	24	7	16	63
80th percentile waiting times (days)	165	80	88	16	889
Non-electives					
Number of patients	166	43,050	24,326	201	133,463
Mean 30-day post discharge survival rate	166	0.95	0.02	0.83	1.00
Mean life expectancy in years	166	34	7	18	65
Outpatient					
Volume of activity	166	427,168	231,608	33,495	1,125,545
*Hospital inputs (£000)*					
NHS labour	166	164,152	98,377	10,030	567,131
Agency labour	166	7,830	6,766	0	47,241
Intermediate goods and services	166	60,907	46,145	6,664	261,787
Capital	166	20,705	14,670	380	84,818

**Table 3:** Descriptive statistics and variable definitions, 2008/09 and 2009/10

Variables	Description	Source	2008/09			2009/10		
			Obs	Mean	SD	Obs	Mean	SD
*Spec*	Percentage of patients receiving specialised care	DH	169	9.69	11.39	166	10.42	12.16
*Specialist_only*	1 if trust is a specialist trust without FT status, 0 otherwise	DH	169	0.04	0.20	166	0.02	0.15
*FT_only*	1 if trust is a non-specialist trust with FT status, 0 otherwise	DH	169	0.41	0.49	166	0.44	0.50
*Specialist_FT*	1 if trust is a specialist trust with FT status, 0 otherwise	DH	169	0.08	0.27	166	0.10	0.30
*Education_p*	Income from education, training and research as proportion of total income	Derived from DH and Monitor	169	5.25	2.9	166	5.21	2.85
*Emerg_p*	Proportion of emergency patients	Derived from HES	169	33.1	10.54	166	33.26	10.57
*Female_p*	Proportion of female patients	Derived from HES	169	56.08	5.61	166	55.89	5.58
*Age_015p*	Proportion of patients under 15 years of age	Derived from HES	169	14.26	13.67	166	14.06	13.7
*Age_1645p*	Proportion of patients between 16 and 45 years of age	Derived from HES	169	29.51	7.94	166	29.12	7.84
*Age_4660p*	Proportion of patients between 46 and 60 years of age	Derived from HES	169	16.79	4.63	166	16.76	4.57
*Age_over60p*	Proportion of patients over 60 years of age	Derived from HES	169	39.44	10.4	166	40.06	10.61
*LoS*	Total inpatient days/total inpatient patients	Derived from HES	169	2.91	0.64	166	2.84	0.64
*Occuppc*	Occupancy rate	DH	169	84.96	6.28	166	84.75	6.41

*DH* Department of Health, *HES* Hospital Episode Statistics

## Results

### Hospital productivity

Productivity ratios and ranks for each hospital are provided in the accompanying spreadsheet (see online supporting material). Our preferred ranking is based on the measure of total factor productivity where we account for quality of hospital care (Eq. 2). Allowing for quality, output is scaled up by an average of 0.46 % in 2008/09 and by 0.37 % in 2009/10, these adjustments being of a similar magnitude to those in the national accounts [11]. But there is wide variation among hospitals in the impact of this adjustment, ranging from more than −6 % at the 5th percentile to more than 10 % at the 95th percentile.

The main points of note are the following:

- We find a substantial variation in hospital productivity, ranging from +45 % above to −62 % below the national average in 2008/09 and from +33 % above to −57 % below the national average in 2009/10.
- The position of individual hospitals does not vary greatly from one year to the next. The correlation between the hospital rankings across the two years is high at *r* = 0.87.
- Productivity scores are not particularly sensitive to whether or not we account for quality. The correlation between the hospital rankings from *P*_*h*_ and from *P*_*h*_^*C*^ is high at *r* = 0.92 and *r* = 0.93, respectively in 2008/09 and 2009/10.
- The Mid Essex Hospital Services NHS Trust emerges as the most productive hospital in 2008/09 and is third most productive in 2009/10.
- At the other end of the spectrum, the same four hospitals have the lowest productivity each year. Three of these are specialist cancer hospitals (The Royal Marsden NHS Foundation Trust, The Christie NHS Foundation Trust, Clatterbridge Centre for Oncology NHS Foundation Trust) and the other is the specialist Royal National Hospital for Rheumatic Diseases NHS Foundation Trust.
- Consider the hospitals that merged. In 2008/09, Queen Elizabeth Hospital NHS Trust was ranked 106/169, Bromley Hospitals NHS Trust 2/169 and Queen Mary’s Sidcup NHS Trust 73/169. The merged hospital (South London Healthcare NHS Trust) was ranked 14/166 in 2009/10. Overall, year-on-year output increased by 7.8 % and input by 7.2 %.
- Similarly, in 2008/09 Worthing and Southlands Hospitals NHS Trust was ranked 75/169 and Royal West Sussex NHS Trust 26/169, while the merged hospital Western Sussex Hospitals NHS Trust was ranked 21/166 in 2009/10. Output increased by 10.7 % and input by 8.8 %.

**Fig. 1:**
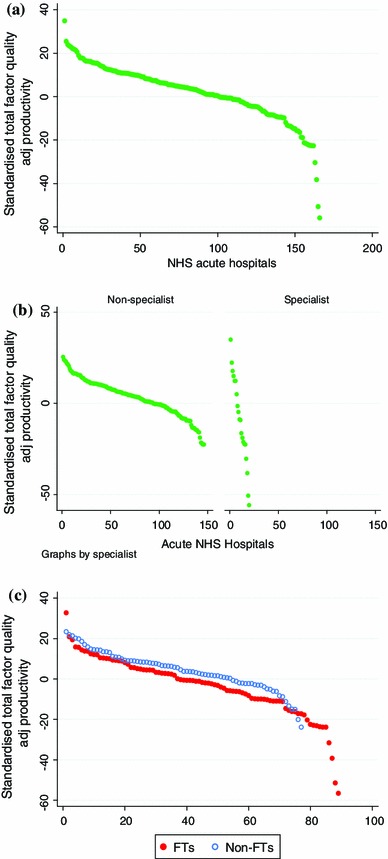
Standardised total factor quality adjusted productivity in 2009/10 for: **a** all NHS acute hospitals, **b** non-specialist versus specialist and **c** by FT status

Figure 1 plots our measure of total factor productivity in 2009/10 based on Eq. 2 for (a) all NHS acute hospitals, (b) by whether or not the hospital is considered specialist and (c) split by FT status. All three graphs show the four outliers with substantially lower productivity than the others, these being The Royal Marsden NHS Foundation Trust, Royal National Hospital for Rheumatic Diseases NHS Foundation Trust, The Christie NHS Foundation Trust, and the Clatterbridge Centre for Oncology NHS Foundation Trust. Graph (b) confirms that specialist hospitals are subject to the widest variation in productivity, suggesting that the ‘specialist’ label is being used to describe a very heterogeneous group of hospitals. Graph (c) shows that FTs tend to have lower productivity than non-FTs, even if we ignore the four hospitals at the bottom of the distribution (which are both specialist hospitals and FTs).

### Variation in hospital productivity

Table 4 presents the results of the OLS regression analysis applied to Eq. (9) for each year. Results for a number of variables are qualitatively consistent across models and between years. First, we find no statistically significant relation between productivity and the proportion of each hospital’s patients that received some form of specialised care, perhaps unsurprisingly given the heterogeneity among specialist hospitals observed in graph (b). Second, FTs tend to be significantly less productive than non-FTs. The difference is driven by the higher expenditure on capital inputs by FTs than by non-FTs—this significance disappears if we consider labour productivity rather than total factor productivity. This implies that FTs are using their borrowing freedoms to invest in their infrastructure but that this investment has not yet yielded a proportionate increase in output. Third, productivity decreases significantly with the proportion of income spent on education, training, and research activities. Finally, hospitals that treat high proportions of both younger (<15) and older (>46) patients tend to have lower productivity than those treating a greater proportion of those in the reference age category (16–45).

**Table 4:** OLS regression of hospital productivity ratios, 2008/09 and 2009/10

	2008/09		2009/10	
	TFP c-adj	TFP q-adj	TFP c-adj	TFP q-adj
	*P*_*h*_^*c*^	*P*_*h*_	*P*_*h*_^*c*^	*P*_*h*_
*Spec*	−0.0000494	−0.0693	−0.0793	−0.0979
	(−0.00)	(−0.52)	(−0.48)	(−0.63)
*Specialist_only*	10.02	16.49**	−1.901	6.626
	(1.30)	(2.36)	(−0.18)	(0.69)
*FT_only*	−5.828***	−5.363***	−6.630***	−6.252***
	(−3.50)	(−3.27)	(−4.68)	(−4.56)
*Specialist_FT*	−15.72**	−12.96*	−14.00*	−12.15*
	(−2.01)	(−1.88)	(−1.78)	(−1.70)
*Education_p*	−2.514***	−2.062***	−2.569***	−2.207***
	(−5.87)	(−5.11)	(−6.39)	(−5.94)
*Emerg_p*	−0.186	−0.177	−0.161	−0.137
	(−1.15)	(−1.12)	(−0.85)	(−0.77)
*Age015p*	−0.779***	−0.831***	−0.697**	−0.765***
	(−3.02)	(−3.41)	(−2.42)	(−2.91)
*Age4660p*	−1.959***	−1.677***	−1.399*	−1.052
	(−3.23)	(−2.87)	(−1.79)	(−1.49)
*Ageover60p*	−0.223	−0.637***	−0.242	−0.670***
	(−1.06)	(−3.45)	(−1.14)	(−3.74)
*Femalep100*	−0.390	−0.736**	−0.481	−0.810**
	(−1.03)	(−2.09)	(−1.04)	(−2.00)
*Occuppc_100*	0.484***	0.474***	0.320*	0.331*
	(2.81)	(2.87)	(1.69)	(1.86)
*LoS*	−6.313**	−7.034***	−5.571*	−6.621**
	(−2.41)	(−2.89)	(−1.83)	(−2.47)
Constant	74.49*	106.5***	82.42	112.1**
	(1.94)	(3.10)	(1.65)	(2.54)
*N*	169	169	166	166
*R* ^2^	0.591	0.507	0.599	0.513

*t*-Statistics in parentheses: * *p* < 0.10; ** *p* < 0.05; *** *p* < 0.01

Productivity does not appear to be related to the proportion of emergency activity. A negative association is found between the proportion of female patients and productivity measured using *P*_*h*_; but when the *P*_*h*_^*C*^ index is used, this significance disappears.

The significance of those variables that capture efficiency in resource use varies from one year to the next. We find that higher rates of occupied beds are associated with higher productivity scores in 2008/09 but that this variable is less statistically significant in 2009/10. Longer average length of stay is sometimes found to be associated with lower productivity, but this relation is only highly significant (*p* < 0.01) when productivity is measured using *P*_*h*_ in 2008/09.

### Variation in hospital outputs

Table 5 presents the results of our regression analysis applied to Eq. (10) for both years. Results are consistent for both measures of our dependent variable and across the two years. The coefficients of labour and capital are positive and statistically significant. This implies that labour and capital have a positive association with output, whether or not quality is accounted for. The coefficient for intermediate input is never significant. We find that the sum of the estimated coefficients for labour (*γ*_1_), capital (*γ*_2_) and intermediate (*γ*_3_) inputs is roughly equal to 0.99 in both years, which suggests that the assumption of constant returns to scale is realistic.

**Table 5:** OLS regressions of hospital output based on choice of functional form, 2008/09 and 2009/10

	2008/9				2009/10			
	Cost adj output	Cost adj output	Quality adj output	Quality adj output	Cost adj output	Cost adj output	Quality adj output	Quality adj output
	*X*_*h*_^*c*^	*X*_*h*_^*c*^	*X*_*h*_	*X*_*h*_	*X*_*h*_^*c*^	*X*_*h*_^*c*^	*X*_*h*_	*X*_*h*_
ln (Labour)	0.933***	0.781***	0.890***	0.772***	0.947***	0.765***	0.880***	0.746***
	(8.22)	(10.86)	(8.47)	(11.97)	(9.82)	(9.27)	(9.78)	(10.47)
ln (Capital)	0.0848**	0.106***	0.0648*	0.106***	0.0878**	0.140***	0.0700**	0.134***
	(2.03)	(3.47)	(1.78)	(3.60)	(2.44)	(4.79)	(2.18)	(5.06)
ln (Intermediate)	−0.0266	0.0822	0.0355	0.0927**	−0.0395	0.0667	0.0443	0.0938
	(−0.37)	(1.58)	(0.54)	(2.04)	(−0.56)	(0.91)	(0.70)	(1.50)
*Spec*		−0.000896		−0.00128		−0.00222		−0.00192
		(−0.62)		(−1.07)		(−1.42)		(−1.39)
*Specialist_only*		−0.0495		0.000860		−0.194*		−0.110
		(−0.53)		(0.01)		(−1.87)		(−1.22)
*FT_only*		−0.0494**		−0.0492**		−0.0735***		−0.0718***
		(−2.00)		(−2.09)		(−3.69)		(−3.92)
*Specialist_FT*		−0.274***		−0.268***		−0.281***		−0.269***
		(−2.69)		(−2.97)		(−2.94)		(−3.14)
*Education_p*		−0.0218***		−0.0174***		−0.0205***		−0.0175***
		(−4.81)		(−4.09)		(−4.97)		(−4.59)
*Emerg_p*		−0.00613***		−0.00600***		−0.00598***		−0.00533***
		(−3.31)		(−3.54)		(−2.98)		(−2.96)
*Age015p*		−0.0109***		−0.00996***		−0.00904***		−0.00858***
		(−3.90)		(−4.09)		(−3.14)		(−3.27)
*Age4660p*		−0.0268***		−0.0209***		−0.0178**		−0.0122*
		(−4.69)		(−3.84)		(−2.29)		(−1.86)
*Ageover60p*		−0.00690***		−0.0106***		−0.00702***		−0.0107***
		(−2.96)		(−5.24)		(−3.06)		(−5.51)
*Femalep*		−0.0114***		−0.0137***		−0.0109***		−0.0131***
		(−3.07)		(−4.20)		(−2.74)		(−3.86)
*Occuppc*		0.00636***		0.00568***		0.00420*		0.00390*
		(3.58)		(3.42)		(1.91)		(1.97)
ln(LoS)		−0.0705		−0.105**		−0.0867**		−0.123***
		(−1.40)		(−2.53)		(−1.99)		(−3.40)
Constant	0.00852	1.854***	0.0481	2.082***	−0.0803	1.864***	−0.0156	2.050***
	(0.02)	(3.47)	(0.12)	(4.36)	(−0.22)	(3.46)	(−0.04)	(4.24)
*N*	169	169	169	169	166	166	166	166
*R* ^2^	0.935	0.970	0.947	0.974	0.944	0.974	0.957	0.978

*t*-Statistics in parentheses: * *p* < 0.10; ** *p* < 0.05; *** *p* < 0.01

Most of the other explanatory variables have a negative impact on output in both years. The exceptions are the proportion of specialised care where the negative influence is modest in magnitude and statistically significant only in 2009/10; the occupancy rate which has a significant positive influence (although only weakly so in 2009/10); and LoS which is generally not highly significant, except in explaining quality-adjusted output in 2009/10.

## Conclusions

The voluminous literature that applies DEA to the hospital sector has had virtually no impact on policy, probably largely because the intended audience lacks confidence in the analytical approach and, hence, the reliability of the results [3]. However, the fundamental policy questions remain: what productivity variation pervades the hospital sector and what are the opportunities for productivity improvement? In this paper we have attempted to address these questions by going back to basics, as advocated by some commentators [40].

Our approach has been to draw on the growth accounting literature used to measure changes in productivity *growth* over time at national or sectoral level and apply this to a cross-sectional context where interest lies in comparing productivity *differences* across organisations within a sector, this being the focus of many DEA studies. Growth indices use weights in the base (Laspeyres index) or current (Paasche index) period. Instead, here we apply weights that reflect the national average for the year in question. Of course, it would be straightforward to extend our formulation to calculate differential productivity growth for each hospital by using as weights the national average costs in the base (Laspeyres) or current (Paasche) period or the geometric mean of the two (Fisher).

Just as in DEA, we define productivity as the ratio of outputs to inputs. However, unlike in DEA, we are explicit about what weight to attach to each type of output so that these can be combined into a single index. In contrast, DEA weights are usually allowed to vary freely, with the maximisation function designed to evaluate each organisation in the best possible light. This might be desirable in some contexts, notably where (1) organisations are free to pursue whatever objectives they choose and (2) where the range of outputs is quite limited. Neither condition holds in the situation that we evaluate. Hospitals in England, and most other countries, are tasked to pursue social objectives, so weights ought to reflect social values [41]. Hospitals also produce a large and diverse range of outputs. In England, as in many countries, there are considerably more outputs than the total number of hospitals, rendering DEA practically unfeasible.

Rankings of relative productivity are sensitive to the choice of weights. To illustrate, the correlation in rankings from our approach and from a DEA model in which inputs are disaggregated into labour, capital and intermediate categories amounts to *r* = 0.84 (2008/09) and *r* = 0.9 (2009/10). The divergence is due to allowing differential weights among organisations in how DEA re-aggregates inputs into a single index. But differential weights are unnecessary in this study because they are measured in monetary units that permit natural aggregation.

If weights are available, DEA is unnecessary: the analytical problem is reduced to construction of a productivity ratio of an output index over an input index [40]. The challenge is to find an appropriate set of weights. In this study, we apply output weights based on observed average costs. These can be defended on two grounds. First, this type of weighting is used in the construction of health productivity measures for the national accounts [42]. Second, the prices that hospitals are paid for their activity are also based on average observed costs [43]. The drawback is that costs, of course, reflect producer rather than consumer valuations [44]. In recognition of this, we incorporate measures of quality into the output index as well. If a comprehensive set of social values was available, allowing a social weight to be attached to each output, it would be straightforward to substitute these for the weights we have employed. This is not, however, an immediate prospect.

A further complaint about DEA is that it does not allow for data error, unlike regression analysis or stochastic frontier analysis (SFA). Of course, it is possible to estimate an SFA version of the Cobb-Douglas production function in which the SFA “inefficiency” term captures all except the classically distributed error variance that is not accounted for in the relationship between inputs and outputs (including those variables included in our *hospvars* vector). SFA estimation of Eq. (10) suggests mean “inefficiency” of around 11–13 % among hospitals, but only if our *hospvars* are included. With the reduced specification no inefficiency term is estimated because the composite error term is normally distributed.

We believe that errors are unlikely to be substantial in the datasets we employ. Our measures of output and quality are constructed from the Hospital Episode Statistics which is subject to various validation and cleaning processes by the NHS Information Authority before it is made available for research purposes. We construct cost weights from cost data reported by all English hospitals, the same data being used to calculate the HRG prices according to which hospitals are paid. Costing errors by individual hospitals will have limited impact because we construct weights based on national average costs. The measures of input are derived from expenditure data reported in each hospital’s audited financial accounts.

Any data errors remaining undetected are unlikely to explain the substantial variations in productivity that we have identified across hospitals in England. We find that hospital productivity varies from +45 % above to −62 % below the national average in 2008/09 and from +33 % to −57 % in 2009/10. For individual hospitals, relative productivity does not vary dramatically year-on-year. Those organisations that merged between the two years exhibited increased productivity.

Some of the variation might be explained by the characteristics of hospitals, and we explored this possibility by estimating regression models with both the productivity ratios and output indices as dependent variables. We find that, just as hospitals labelled as ‘specialist’ appear very heterogeneous in terms of their productivity, the proportion of patients receiving specialist services does not explain variation in productivity significantly. Hospitals that have been granted Foundation Trust status tend to be less productive and have lower output than non-FTs, which may reflect lags in benefit realisation following capital investment. Finally, we find evidence of a negative and significant association between productivity and the proportion of income from education, research and development, and training activities.

Our analyses suggest substantial scope for productivity improvement across the English hospital sector. It would be worth focusing attention on those hospitals at the top and bottom of the rankings in order to identify specific drivers of differential productivity in those organisations.

## Electronic supplementary material

Below is the link to the electronic supplementary material.

Supplementary material 1 (PDF 285 kb)

Supplementary material 2 (PDF 282 kb)

## Footnotes

1 We are grateful to one of the referees for guidance in arriving at this formulation.

2 The following mergers took place in 2009/10. Queen Elizabeth Hospital NHS Trust (RG2), Bromley Hospitals NHS Trust (RG3) and Queen Mary’s Sidcup NHS Trust (RGZ) merged to become South London Healthcare NHS Trust (RYQ), while Worthing and Southlands Hospitals NHS Trust (RPL) and Royal West Sussex NHS Trust (RPR) merged to form Western Sussex Hospitals NHS Trust (RYR).

3 For one hospital, the Lewisham Hospital NHS Trust, all values for waiting times are missing.
