# Supplementary material for: Examining variations in hospital productivity in the English NHS
Source: Eur J Health Econ. 2014 Feb 25;16(3):243–54. doi: 10.1007/s10198-014-0569-5 (PMC4361750; doi:10.1007/s10198-014-0569-5)
Supplement: Supplementary file 1 — Supplementary material 1 (PDF 285 kb) [file 10198_2014_569_MOESM1_ESM.pdf]

2008/09 Hospital Trust*	Provider code	Cost adjusted total outputs (Inpatient and Outpatient)	Quality adjusted total outputs (Inpatient) & Cost adjusted total outputs (outpatient)	Total NHS inputs (expenditure)	TFP cost-adj		TFP q-adj	
					Standardised score	Ranking	Standardised score	Ranking
Mid Essex Hospital Services NHS Trust	RQ8	145,558	141,621	182,003	48.93	1	45.01	1
Liverpool Women's NHS Foundation Trust	REP	55,382	56,529	81,610	26.38	4	29.09	2
Bromley Hospitals NHS Trust	RG3	101,704	97,311	142,887	32.55	2	26.92	3
Queen Victoria Hospital NHS Foundation Trust	RPC	30,818	31,711	47,633	20.48	11	24.07	4
The Royal Orthopaedic Hospital NHS Foundation Trust	RRJ	38,059	40,723	61,445	15.35	25	23.51	5
The Queen Elizabeth Hospital King's Lynn NHS Trust	RCX	87,808	83,300	125,964	29.81	3	23.24	6
Robert Jones and Agnes Hunt Orthopaedic and District Hospital NHS Trust	RL1	38,463	40,376	61,146	17.14	18	23.06	7
Mayday Healthcare NHS Trust	RJ6	95,623	96,350	146,057	21.92	8	22.94	8
Southampton University Hospitals NHS Trust	RHM	264,306	265,865	403,327	22.03	7	22.85	9
The Royal Wolverhampton Hospitals NHS Trust	RL4	156,784	153,444	236,016	23.71	6	21.16	10
Liverpool Heart and Chest Hospital NHS Trust	RBQ	45,468	46,300	71,299	18.76	14	21.02	11
South Warwickshire General Hospitals NHS Trust	RJC	67,119	63,988	98,933	26.34	5	20.54	12
Royal Cornwall Hospitals NHS Trust	REF	173,453	168,330	267,895	20.57	10	17.10	13
The Princess Alexandra Hospital NHS Trust	RQW	80,043	77,699	124,631	19.60	13	16.19	14
West Suffolk Hospitals NHS Trust	RGR	81,509	78,282	125,567	20.88	9	16.18	15
Bedford Hospital NHS Trust	RC1	67,875	67,423	108,307	16.70	20	16.02	16
Warrington and Halton Hospitals NHS Foundation Trust	RWW	100,487	103,493	166,395	12.46	30	15.91	17
The Dudley Group of Hospitals NHS Foundation Trust	RNA	137,354	132,917	213,757	19.66	12	15.88	18
Great Western Hospitals NHS Foundation Trust	RN3	106,414	106,574	171,412	15.61	23	15.87	19
Whipps Cross University Hospital NHS Trust	RGC	102,792	102,376	165,088	15.95	22	15.57	20
Hinchingbrooke Health Care NHS Trust	RQQ	47,305	46,419	75,162	17.20	17	15.10	21
Milton Keynes Hospital NHS Foundation Trust	RD8	78,591	80,266	130,611	12.05	33	14.53	22
Chelsea and Westminster Hospital NHS Foundation Trust	RQM	127,374	134,713	219,503	8.06	46	14.38	23
Ealing Hospital NHS Trust	RC3	62,041	65,951	108,075	6.90	49	13.73	24
West Hertfordshire Hospitals NHS Trust	RWG	119,016	114,297	187,787	18.02	15	13.43	25
The Hillingdon Hospital NHS Trust	RAS	80,792	81,499	134,169	12.14	32	13.20	26
Royal Berkshire NHS Foundation Trust	RHW	153,248	152,440	251,121	13.64	28	13.13	27
George Eliot Hospital NHS Trust	RLT	54,767	52,623	86,717	17.61	16	13.09	28
Wrightington, Wigan and Leigh NHS Foundation Trust	RRF	118,539	120,660	200,336	10.19	40	12.25	29
Ashford and St Peter's Hospitals NHS Trust	RTK	104,165	101,245	168,136	15.37	24	12.22	30
Newham University Hospital NHS Trust	RNH	72,940	76,855	127,791	6.29	53	12.08	31
Surrey and Sussex Healthcare NHS Trust	RTP	91,909	87,984	147,158	16.31	21	11.42	32
Royal Brompton and Harefield NHS Trust	RT3	104,189	113,541	190,714	1.74	85	10.95	33
Royal National Orthopaedic Hospital NHS Trust	RAN	29,058	36,597	61,899	-12.58	145	10.19	34
Pennine Acute Hospitals NHS Trust	RW6	279,410	278,646	471,488	10.36	39	10.14	35
Frimley Park Hospital NHS Foundation Trust	RDU	110,115	110,678	187,416	9.41	43	10.06	36
Southport and Ormskirk Hospital NHS Trust	RVY	78,923	75,832	128,453	14.42	27	10.02	37
South Tees Hospitals NHS Trust	RTR	222,263	225,102	383,916	7.81	47	9.27	38
Heart of England NHS Foundation Trust	RR1	284,859	282,928	484,058	9.59	42	8.93	39
Peterborough and Stamford Hospitals NHS Foundation Trust	RGN	104,865	105,387	181,144	7.81	48	8.42	40
Luton and Dunstable Hospital NHS Foundation Trust	RC9	98,831	101,111	174,258	5.62	60	8.14	41
Northampton General Hospital NHS Trust	RNS	105,375	107,287	184,935	6.11	54	8.12	42
East Lancashire Hospitals NHS Trust	RXR	168,160	174,543	301,033	4.03	72	8.06	43
St Helens and Knowsley Teaching Hospitals NHS Trust	RBN	113,673	115,644	199,619	6.04	55	7.97	44

2008/09 Hospital Trust*	Provider code	Cost adjusted total outputs (Inpatient and Outpatient)	Quality adjusted total outputs (Inpatient) & Cost adjusted total outputs (outpatient)	Total NHS inputs (expenditure)	TFP cost-adj		TFP q-adj	
					Standardised score	Ranking	Standardised score	Ranking
Royal West Sussex NHS Trust	RPR	72,299	67,905	117,333	14.75	26	7.86	45
West Middlesex University Hospital NHS Trust	RFW	66,081	67,956	117,630	4.62	69	7.66	46
Barnet and Chase Farm Hospitals NHS Trust	RVL	139,405	134,699	233,491	11.18	34	7.51	47
James Paget University Hospitals NHS Foundation Trust	RGP	94,649	86,811	150,582	17.05	19	7.44	48
Gloucestershire Hospitals NHS Foundation Trust	RTE	233,448	226,240	393,148	10.58	36	7.24	49
North Middlesex University Hospital NHS Trust	RAP	64,599	68,327	118,771	1.29	90	7.21	50
United Lincolnshire Hospitals NHS Trust	RWD	204,806	193,285	336,075	13.49	29	7.18	51
Kettering General Hospital NHS Foundation Trust	RNQ	88,378	85,425	149,033	10.43	37	6.82	52
Tameside Hospital NHS Foundation Trust	RMP	72,150	72,733	127,148	5.67	59	6.61	53
North Bristol NHS Trust	RVJ	215,292	214,669	375,753	6.70	50	6.47	54
Dartford and Gravesham NHS Trust	RN7	65,837	62,158	109,236	12.24	31	6.05	55
Countess of Chester Hospital NHS Foundation Trust	RJR	92,495	91,795	161,518	6.64	51	5.92	56
Walsall Hospitals NHS Trust	RBK	86,252	86,597	153,180	4.86	67	5.36	57
Lancashire Teaching Hospitals NHS Foundation Trust	RXN	193,270	194,368	344,197	4.57	70	5.24	58
University Hospital of North Staffordshire NHS Trust	RJE	198,019	198,172	351,368	4.95	65	5.11	59
St George's Healthcare NHS Trust	RJ7	178,390	184,505	327,643	1.39	87	4.95	60
Worcestershire Acute Hospitals NHS Trust	RWP	157,896	152,751	271,843	8.16	44	4.72	61
Kingston Hospital NHS Trust	RAX	85,980	82,436	148,098	8.11	45	3.74	62
Mid Cheshire Hospitals NHS Foundation Trust	RBT	85,449	83,593	150,574	5.68	58	3.46	63
Heatherwood and Wexham Park Hospitals NHS Foundation Trust	RD7	111,027	110,763	199,565	3.60	76	3.44	64
Shrewsbury and Telford Hospital NHS Trust	RXW	128,835	127,162	229,149	4.70	68	3.42	65
The Rotherham NHS Foundation Trust	RFR	92,163	92,201	166,528	3.06	78	3.18	66
Plymouth Hospitals NHS Trust	RK9	193,230	192,274	347,362	3.59	77	3.16	67
Nuffield Orthopaedic Centre NHS Trust	RBF	26,920	27,937	50,550	-0.83	103	3.00	68
Colchester Hospital University NHS Foundation Trust	RDE	107,582	100,063	181,538	10.36	38	2.72	69
Winchester and Eastleigh Healthcare NHS Trust	RN1	68,899	67,162	121,958	5.20	64	2.63	70
East Cheshire NHS Trust	RJN	57,775	55,789	101,722	5.77	57	2.21	71
East Sussex Hospitals NHS Trust	RXC	142,548	132,342	241,338	9.99	41	2.20	72
Burton Hospitals NHS Foundation Trust	RJF	69,307	67,453	123,457	4.54	71	1.82	73
Homerton University Hospital NHS Foundation Trust	RQX	70,261	74,561	136,712	-4.29	117	1.64	74
Buckinghamshire Hospitals NHS Trust	RXQ	133,170	130,144	238,892	3.81	74	1.53	75
Doncaster and Bassetlaw Hospitals NHS Foundation Trust	RP5	161,469	161,735	297,683	1.01	92	1.25	76
Stockport NHS Foundation Trust	RWJ	108,760	107,201	197,564	2.52	81	1.12	77
Royal Bolton Hospital NHS Foundation Trust	RMC	94,203	94,744	174,820	0.35	95	1.00	78
Basingstoke and North Hampshire NHS Foundation Trust	RN5	72,428	71,599	132,341	1.92	84	0.83	79
Hereford Hospitals NHS Trust	RLQ	57,508	54,984	101,718	5.28	63	0.74	80
Bradford Teaching Hospitals NHS Foundation Trust	RAE	146,757	153,473	284,079	-3.80	116	0.68	81
Ipswich Hospital NHS Trust	RGQ	107,407	101,288	187,616	6.61	52	0.61	82
Royal Devon and Exeter NHS Foundation Trust	RH8	168,646	165,158	306,387	2.50	82	0.46	83
Basildon and Thurrock University Hospitals NHS Foundation Trust	RDD	120,382	117,286	218,060	2.81	79	0.24	84
Dorset County Hospital NHS Foundation Trust	RBD	80,642	76,439	142,289	5.54	61	0.12	85
County Durham and Darlington NHS Foundation Trust	RXP	165,546	165,863	309,781	-0.48	98	-0.22	86
University Hospitals of Leicester NHS Trust	RWE	308,135	318,853	596,267	-3.77	115	-0.34	87
Chesterfield Royal Hospital NHS Foundation Trust	RFS	87,428	84,979	158,968	2.42	83	-0.38	88

2008/09 Hospital Trust*	Provider code	Cost adjusted total outputs (Inpatient and Outpatient)	Quality adjusted total outputs (Inpatient) & Cost adjusted total outputs (outpatient)	Total NHS inputs (expenditure)	TFP cost-adj		TFP q-adj	
					Standardised score	Ranking	Standardised score	Ranking
Portsmouth Hospitals NHS Trust	RHU	198,575	198,387	372,133	-0.63	100	-0.65	89
Norfolk and Norwich University Hospitals NHS Foundation Trust	RM1	244,994	231,701	434,761	4.94	66	-0.68	90
Sandwell and West Birmingham Hospitals NHS Trust	RXK	174,263	178,954	336,512	-3.56	113	-0.89	91
The Whittington Hospital NHS Trust	RKE	64,184	69,265	130,295	-8.27	133	-0.93	92
Moorfields Eye Hospital NHS Foundation Trust	RP6	42,714	46,850	88,155	-9.77	135	-0.96	93
University Hospitals of Morecambe Bay NHS Trust	RTX	123,631	119,052	224,375	2.61	80	-1.12	94
Derby Hospitals NHS Foundation Trust	RTG	198,835	195,328	368,354	0.52	94	-1.18	95
University Hospitals Coventry and Warwickshire NHS Trust	RKB	203,235	202,536	382,044	-0.94	104	-1.20	96
Queen Mary's Sidcup NHS Trust	RGZ	54,744	51,992	98,162	3.85	73	-1.29	97
Alder Hey Children's NHS Foundation Trust	RBS	72,929	82,069	154,957	-12.36	144	-1.30	98
Salisbury NHS Foundation Trust	RNZ	89,757	88,668	167,700	-0.33	97	-1.46	99
Royal United Hospital Bath NHS Trust	RD1	95,817	93,016	176,112	1.32	89	-1.57	100
Epsom and St Helier University Hospitals NHS Trust	RVR	131,548	127,296	241,117	1.60	86	-1.61	101
Scarborough and North East Yorkshire Health Care NHS Trust	RCC	56,165	52,158	98,811	5.85	56	-1.63	102
University Hospitals Bristol NHS Foundation Trust	RA7	204,401	217,754	413,575	-7.96	131	-1.88	103
Airedale NHS Trust	RCF	59,702	57,745	109,713	1.34	88	-1.91	104
Central Manchester University Hospitals NHS Foundation Trust	RW3	264,489	297,021	565,208	-12.86	146	-2.06	105
Mid Yorkshire Hospitals NHS Trust	RXF	180,131	177,297	337,530	-0.62	99	-2.11	106
Nottingham University Hospitals NHS Trust	RX1	302,173	309,920	590,461	-4.70	121	-2.18	107
Medway NHS Foundation Trust	RPA	92,747	93,317	177,850	-2.89	109	-2.22	108
Barnsley Hospital NHS Foundation Trust	RFF	76,828	76,571	145,955	-1.98	107	-2.23	109
Mid Staffordshire NHS Foundation Trust	RJD	76,507	74,476	141,990	0.34	96	-2.25	110
Oxford Radcliffe Hospitals NHS Trust	RTH	271,259	277,524	529,545	-4.61	119	-2.33	111
Royal Liverpool and Broadgreen University Hospitals NHS Trust	RQ6	159,607	168,856	322,210	-7.75	129	-2.33	112
Blackpool, Fylde and Wyre Hospitals NHS Foundation Trust	RXL	141,363	130,907	249,854	5.36	62	-2.36	113
City Hospitals Sunderland NHS Foundation Trust	RLN	142,684	143,787	275,175	-3.44	112	-2.62	114
Royal Surrey County Hospital NHS Trust	RA2	86,379	84,367	162,165	-0.81	102	-3.04	115
The Lewisham Hospital NHS Trust	RJ2	70,683	72,140	138,701	-5.10	124	-3.07	116
The Royal Bournemouth and Christchurch Hospitals NHS Foundation Trust	RDZ	123,999	108,385	208,418	10.79	35	-3.08	117
Southend University Hospital NHS Foundation Trust	RAJ	112,811	109,921	211,445	-0.65	101	-3.12	118
Queen Elizabeth Hospital NHS Trust	RG2	71,693	70,572	135,784	-1.68	106	-3.14	119
Poole Hospital NHS Foundation Trust	RD3	95,650	91,343	175,959	1.23	91	-3.26	120
Aintree University Hospitals NHS Foundation Trust	REM	127,473	125,126	241,292	-1.62	105	-3.36	121
Worthing and Southlands Hospitals NHS Trust	RPL	87,349	80,725	156,699	3.81	75	-3.99	122
Yeovil District Hospital NHS Foundation Trust	RA4	52,222	49,635	96,444	0.84	93	-4.09	123
East Kent Hospitals University NHS Foundation Trust	RVV	209,734	201,999	399,570	-2.25	108	-5.79	124
Hull and East Yorkshire Hospitals NHS Trust	RWA	210,458	207,887	412,110	-4.90	123	-5.99	125
Barking, Havering and Redbridge Hospitals NHS Trust	RF4	178,727	173,081	345,325	-3.62	114	-6.59	126
North Cumbria University Hospitals NHS Trust	RNL	105,184	102,408	205,531	-4.70	120	-7.14	127
North West London Hospitals NHS Trust	RV8	131,512	136,005	273,080	-10.32	138	-7.18	128
Taunton and Somerset NHS Foundation Trust	RBA	106,194	101,870	204,740	-3.41	111	-7.27	129
The Walton Centre for Neurology and Neurosurgery NHS Trust	RET	25,072	25,726	51,913	-10.06	137	-7.65	130
Maidstone and Tunbridge Wells NHS Trust	RWF	131,634	127,124	257,608	-4.84	122	-8.03	131
The Newcastle Upon Tyne Hospitals NHS Foundation Trust	RTD	317,232	331,979	673,249	-12.25	143	-8.10	132

2008/09 Hospital Trust[*]	Provider code	Cost adjusted total outputs (Inpatient and Outpatient)	Quality adjusted total outputs (Inpatient) & Cost adjusted total outputs (outpatient)	Total NHS inputs (expenditure)	TFP cost-adj		TFP q-adj	
					Standardised score	Ranking	Standardised score	Ranking
Harrogate and District NHS Foundation Trust	RCD	58,437	55,207	112,154	-2.97	110	-8.26	133
Northumbria Healthcare NHS Foundation Trust	RTF	145,332	141,161	287,173	-5.76	125	-8.39	134
South Devon Healthcare NHS Foundation Trust	RA9	101,247	97,041	197,502	-4.54	118	-8.43	135
King's College Hospital NHS Foundation Trust	RJZ	196,721	212,057	435,992	-15.98	149	-9.36	136
Calderdale and Huddersfield NHS Foundation Trust	RWY	143,942	141,244	290,969	-7.88	130	-9.53	137
Royal Free Hampstead NHS Trust	RAL	177,834	185,519	386,253	-14.26	148	-10.49	138
Northern Lincolnshire and Goole Hospitals NHS Foundation Trust	RJL	136,704	130,998	274,239	-7.17	126	-10.98	139
Trafford Healthcare NHS Trust	RM4	43,164	42,576	89,310	-10.00	136	-11.16	140
Sheffield Children's NHS Foundation Trust	RCU	41,193	49,244	103,444	-25.84	159	-11.28	141
Salford Royal NHS Foundation Trust	RM3	131,148	139,903	294,757	-17.14	151	-11.54	142
Wirral University Teaching Hospital NHS Foundation Trust	RBL	125,474	123,118	260,730	-10.38	139	-12.00	143
Brighton and Sussex University Hospitals NHS Trust	RXH	161,832	160,606	340,139	-11.40	141	-12.00	144
Sherwood Forest Hospitals NHS Foundation Trust	RK5	102,299	102,364	217,094	-12.25	142	-12.13	145
Northern Devon Healthcare NHS Trust	RBZ	58,175	54,324	117,017	-7.42	127	-13.48	146
East and North Hertfordshire NHS Trust	RWH	124,732	116,990	252,729	-8.09	132	-13.73	147
York Hospitals NHS Foundation Trust	RCB	106,309	102,254	221,830	-10.76	140	-14.09	148
Weston Area Health NHS Trust	RA3	38,551	35,669	77,643	-7.54	128	-14.39	149
Papworth Hospital NHS Foundation Trust	RGM	52,048	48,991	107,040	-9.45	134	-14.70	150
South Tyneside NHS Foundation Trust	RE9	45,617	44,989	98,380	-13.65	147	-14.78	151
Birmingham Women's NHS Foundation Trust	RLU	34,819	35,263	77,774	-16.63	150	-15.50	152
Sheffield Teaching Hospitals NHS Foundation Trust	RHQ	324,873	333,260	735,139	-17.70	152	-15.52	153
University Hospital of South Manchester NHS Foundation Trust	RM2	133,956	135,662	303,544	-17.82	153	-16.71	154
Imperial College Healthcare NHS Trust	RYJ	267,038	285,769	650,777	-23.59	157	-18.16	155
Leeds Teaching Hospitals NHS Trust	RR8	332,920	349,626	801,195	-22.62	156	-18.67	156
Birmingham Children's Hospital NHS Foundation Trust	RQ3	63,677	75,482	173,453	-31.63	163	-18.90	157
Gateshead Health NHS Foundation Trust	RR7	71,788	72,870	169,782	-21.26	154	-20.01	158
North Tees and Hartlepool NHS Foundation Trust	RVW	102,991	105,741	247,451	-22.49	155	-20.36	159
Cambridge University Hospitals NHS Foundation Trust	RGT	198,433	205,557	483,736	-23.61	158	-20.81	160
Barts and The London NHS Trust	RNJ	185,183	203,929	484,807	-28.87	161	-21.61	161
Guy's and St Thomas' NHS Foundation Trust	RJ1	252,766	271,501	662,054	-28.90	162	-23.57	162
University Hospitals Birmingham NHS Foundation Trust	RRK	170,841	182,456	446,432	-28.74	160	-23.83	163
Great Ormond Street Hospital For Children NHS Trust	RP4	63,033	83,728	226,096	-48.08	166	-30.99	164
University College London Hospitals NHS Foundation Trust	RRV	169,696	184,068	499,793	-36.77	164	-31.36	165
The Royal Marsden NHS Foundation Trust	RPY	50,452	54,466	166,403	-43.54	165	-39.00	166
Royal National Hospital for Rheumatic Diseases NHS Foundation Trust	RBB	5,018	5,364	18,019	-48.14	167	-44.52	167
The Christie NHS Foundation Trust	RBV	30,748	34,693	144,893	-60.48	168	-55.38	168
Clatterbridge Centre for Oncology NHS Foundation Trust	REN	10,536	11,713	57,602	-65.94	169	-62.10	169

[*] Hospital Trusts have been ordered according to the TFP quality-adj measure
