# Supplementary material for: Examining variations in hospital productivity in the English NHS
Source: Eur J Health Econ. 2014 Feb 25;16(3):243–54. doi: 10.1007/s10198-014-0569-5 (PMC4361750; doi:10.1007/s10198-014-0569-5)
Supplement: Supplementary file 2 — Supplementary material 2 (PDF 282 kb) [file 10198_2014_569_MOESM2_ESM.pdf]

2009/10 Hospital Trust*	Provider code	Cost adjusted total outputs (Inpatient and Outpatient)	Quality adjusted total outputs (Inpatient) & Cost adjusted total outputs (outpatient)	Total NHS inputs (expenditure)	TFP cost-adj		TFP quality-adj	
					Standardised score	Ranking	Standardised score	Ranking
Queen Victoria Hospital NHS Foundation Trust	RPC	33,513	33,798	49,213	31.38	1	32.72	1
East Lancashire Hospitals NHS Trust	RXR	196,858	199,167	311,888	21.77	5	23.41	2
Mid Essex Hospital Services NHS Trust	RQ8	130,166	125,974	199,625	25.80	3	21.95	3
Surrey and Sussex Healthcare NHS Trust	RTP	96,971	93,546	148,946	25.60	4	21.37	4
Great Western Hospitals NHS Foundation Trust	RN3	113,168	112,368	179,670	21.51	6	20.86	5
Robert Jones and Agnes Hunt Orthopaedic and District Hospital NHS Trust	RL1	39,818	41,473	66,665	15.23	18	20.23	6
Mayday Healthcare NHS Trust	RJ6	98,147	99,267	160,015	18.33	12	19.89	7
South Warwickshire General Hospitals NHS Trust	RJC	73,872	69,597	112,624	26.54	2	19.42	8
Newham University Hospital NHS Trust	RNH	77,256	82,756	134,853	10.52	40	18.59	9
Ashford and St Peter's Hospitals NHS Trust	RTK	111,923	107,818	178,012	21.30	7	17.05	10
Dartford and Gravesham NHS Trust	RN7	70,813	68,500	114,214	19.61	8	15.90	11
The Royal Orthopaedic Hospital NHS Foundation Trust	RRJ	37,883	39,290	65,538	11.51	35	15.86	12
Warrington and Halton Hospitals NHS Foundation Trust	RWW	109,714	112,265	187,706	12.76	28	15.58	13
George Eliot Hospital NHS Trust	RLT	59,039	57,006	95,948	18.71	10	14.82	14
The Royal Wolverhampton Hospitals NHS Trust	RL4	157,491	153,762	259,552	17.06	17	14.49	15
Bedford Hospital NHS Trust	RC1	70,394	68,235	115,269	17.82	13	14.40	16
The Dudley Group of Hospitals NHS Foundation Trust	RNA	150,867	145,223	245,361	18.62	11	14.38	17
West Hertfordshire Hospitals NHS Trust	RWG	122,135	118,708	200,814	17.34	15	14.24	18
Frimley Park Hospital NHS Foundation Trust	RDU	118,474	119,309	202,612	12.81	27	13.80	19
Wrightington, Wigan and Leigh NHS Foundation Trust	RRF	127,553	129,500	220,116	11.79	33	13.70	20
West Middlesex University Hospital NHS Trust	RFW	72,017	73,967	125,920	10.34	42	13.52	21
South London Healthcare NHS Trust	RYQ	245,887	237,087	403,881	17.45	14	13.44	22
Royal National Orthopaedic Hospital NHS Trust	RAN	34,113	41,505	70,917	-7.20	125	13.10	23
Milton Keynes Hospital NHS Foundation Trust	RD8	80,102	82,496	141,723	9.04	47	12.49	24
Royal Berkshire NHS Foundation Trust	RHW	153,983	150,412	259,329	14.55	19	12.09	25
Dorset County Hospital NHS Foundation Trust	RBD	90,115	84,610	146,068	19.02	9	11.94	26
The Hillingdon Hospital NHS Trust	RAS	83,617	84,838	147,394	9.45	46	11.23	27
Ealing Hospital NHS Trust	RC3	60,759	63,630	110,911	5.69	61	10.87	28
Barnet and Chase Farm Hospitals NHS Trust	RVL	152,054	146,980	256,599	14.32	22	10.70	29
Liverpool Women's NHS Foundation Trust	REP	48,353	49,349	86,294	8.10	49	10.52	30
Liverpool Heart and Chest Hospital NHS Trust	RBQ	47,296	47,300	82,772	10.24	44	10.43	31
Heart of England NHS Foundation Trust	RR1	302,479	299,860	525,851	10.97	38	10.20	32
Heatherwood and Wexham Park Hospitals NHS Foundation Trust	RD7	122,174	121,542	213,410	10.45	41	10.06	33
West Suffolk Hospitals NHS Trust	RGR	80,855	77,840	136,849	13.98	23	9.92	34
Lancashire Teaching Hospitals NHS Foundation Trust	RXN	205,796	209,044	369,377	7.48	52	9.37	35
Gloucestershire Hospitals NHS Foundation Trust	RTE	235,117	227,683	402,679	12.64	29	9.27	36
Worcestershire Acute Hospitals NHS Trust	RWP	165,278	159,421	282,150	13.01	25	9.19	37
Chelsea and Westminster Hospital NHS Foundation Trust	RQM	130,513	137,512	243,698	3.32	78	9.05	38
The Princess Alexandra Hospital NHS Trust	RQW	83,626	81,169	143,884	12.13	32	9.02	39
Kingston Hospital NHS Trust	RAX	91,522	88,793	157,412	12.17	31	9.01	40
Hinchingbrooke Health Care NHS Trust	RQQ	49,111	47,855	84,889	11.61	34	8.94	41
Countess of Chester Hospital NHS Foundation Trust	RJR	97,738	97,046	172,239	9.47	45	8.89	42
Southport and Ormskirk Hospital NHS Trust	RVY	80,845	76,948	136,858	13.96	24	8.66	43
Royal Cornwall Hospitals NHS Trust	REF	163,695	157,644	280,830	12.45	30	8.48	44
Shrewsbury and Telford Hospital NHS Trust	RXW	143,205	139,561	248,965	10.97	39	8.33	45

2009/10 Hospital Trust*	Provider code	Cost adjusted total outputs (Inpatient and Outpatient)	Quality adjusted total outputs (Inpatient) & Cost adjusted total outputs (outpatient)	Total NHS inputs (expenditure)	TFP cost-adj		TFP quality-adj	
					Standardised score	Ranking	Standardised score	Ranking
Whipps Cross University Hospital NHS Trust	RGC	105,547	105,469	188,171	8.21	48	8.32	46
Norfolk and Norwich University Hospitals NHS Foundation Trust	RM1	228,251	215,033	384,459	14.54	20	8.09	47
Southampton University Hospitals NHS Trust	RHM	233,199	239,648	428,749	4.93	70	8.02	48
Walsall Hospitals NHS Trust	RBK	89,346	89,050	159,618	7.99	50	7.81	49
The Queen Elizabeth Hospital King's Lynn NHS Trust	RCX	83,244	79,287	142,350	12.82	26	7.64	50
Peterborough and Stamford Hospitals NHS Foundation Trust	RGN	111,493	111,270	200,295	7.39	55	7.36	51
University Hospital of North Staffordshire NHS Trust	RJE	215,682	215,012	387,287	7.44	54	7.29	52
Northampton General Hospital NHS Trust	RNS	111,950	113,310	205,467	5.11	68	6.57	53
Western Sussex Hospitals NHS Trust	RYR	176,714	164,318	298,131	14.35	21	6.51	54
Plymouth Hospitals NHS Trust	RK9	197,073	197,181	358,267	6.12	59	6.36	55
Sandwell and West Birmingham Hospitals NHS Trust	RXK	190,687	196,864	357,802	2.82	82	6.33	56
St George's Healthcare NHS Trust	RJ7	195,074	203,324	371,147	1.40	93	5.87	57
The Royal Bournemouth and Christchurch Hospitals NHS Foundation Trust	RDZ	133,466	120,081	219,589	17.26	16	5.68	58
Southend University Hospital NHS Foundation Trust	RAJ	130,344	123,751	226,403	11.07	36	5.63	59
Pennine Acute Hospitals NHS Trust	RW6	282,540	283,139	518,111	5.21	65	5.61	60
Luton and Dunstable Hospital NHS Foundation Trust	RC9	101,475	103,030	189,292	3.42	77	5.19	61
Tameside Hospital NHS Foundation Trust	RMP	74,071	74,086	136,472	4.71	73	4.91	62
Barking, Havering and Redbridge Hospitals NHS Trust	RF4	192,696	187,752	346,917	7.16	56	4.59	63
The Rotherham NHS Foundation Trust	RFR	97,051	96,744	178,796	4.72	72	4.57	64
Colchester Hospital University NHS Foundation Trust	RDE	120,550	113,226	209,420	11.05	37	4.48	65
County Durham and Darlington NHS Foundation Trust	RXP	173,228	172,932	320,083	4.41	75	4.41	66
Salisbury NHS Foundation Trust	RNZ	93,060	91,136	168,820	6.35	58	4.33	67
St Helens and Knowsley Teaching Hospitals NHS Trust	RBN	115,191	117,498	218,701	1.61	91	3.83	68
Winchester and Eastleigh Healthcare NHS Trust	RN1	69,384	66,893	124,537	7.48	53	3.80	69
Hereford Hospitals NHS Trust	RLQ	58,876	56,675	105,639	7.52	51	3.68	70
North Middlesex University Hospital NHS Trust	RAP	65,087	68,629	128,068	-1.95	104	3.56	71
City Hospitals Sunderland NHS Foundation Trust	RLN	154,497	155,077	290,063	2.76	83	3.32	72
Moorfields Eye Hospital NHS Foundation Trust	RP6	48,105	52,730	98,742	-6.01	121	3.20	73
Royal United Hospital Bath NHS Trust	RD1	104,420	100,677	188,543	6.84	57	3.19	74
Kettering General Hospital NHS Foundation Trust	RNQ	92,541	90,714	170,339	4.81	71	2.92	75
Poole Hospital NHS Foundation Trust	RD3	102,713	99,746	187,531	5.67	62	2.79	76
Epsom and St Helier University Hospitals NHS Trust	RVR	140,016	135,815	255,542	5.71	60	2.71	77
Bradford Teaching Hospitals NHS Foundation Trust	RAE	153,033	161,184	303,399	-2.69	110	2.67	78
Buckinghamshire Hospitals NHS Trust	RXQ	133,812	130,155	245,586	5.12	67	2.42	79
Chesterfield Royal Hospital NHS Foundation Trust	RFS	92,700	90,181	170,211	5.07	69	2.39	80
North Bristol NHS Trust	RVJ	219,433	218,663	413,541	2.37	88	2.18	81
University Hospitals Coventry and Warwickshire NHS Trust	RKB	211,533	213,106	403,931	1.03	95	1.96	82
Ipswich Hospital NHS Trust	RGQ	112,773	108,891	206,547	5.33	64	1.88	83
Basingstoke and North Hampshire NHS Foundation Trust	RN5	76,034	75,210	142,800	2.72	84	1.78	84
University Hospitals of Leicester NHS Trust	RWE	329,964	340,589	647,918	-1.75	102	1.59	85
North Cumbria University Hospitals NHS Trust	RNL	109,457	105,520	200,742	5.19	66	1.58	86
University Hospitals of Morecambe Bay NHS Trust	RTX	131,055	125,569	239,782	5.44	63	1.20	87
East Sussex Hospitals NHS Trust	RXC	147,389	134,562	257,910	10.25	43	0.83	88
Hull and East Yorkshire Hospitals NHS Trust	RWA	232,372	227,415	436,956	2.60	85	0.58	89
The Lewisham Hospital NHS Trust	RJ2	73,131	74,603	143,632	-1.77	103	0.38	90

2009/10 Hospital Trust*	Provider code	Cost adjusted total outputs (Inpatient and Outpatient)	Quality adjusted total outputs (Inpatient) & Cost adjusted total outputs (outpatient)	Total NHS inputs (expenditure)	TFP cost-adj		TFP quality-adj	
					Standardised score	Ranking	Standardised score	Ranking
Mid Cheshire Hospitals NHS Foundation Trust	RBT	86,112	84,164	162,531	2.21	89	0.07	91
Barnsley Hospital NHS Foundation Trust	RFF	81,738	81,406	157,902	-0.13	99	-0.37	92
Calderdale and Huddersfield NHS Foundation Trust	RWY	152,779	150,089	291,737	1.03	94	-0.58	93
Royal Surrey County Hospital NHS Trust	RA2	98,010	94,308	183,407	3.09	80	-0.63	94
Doncaster and Bassetlaw Hospitals NHS Foundation Trust	RP5	166,138	164,611	320,375	0.04	98	-0.71	95
East Cheshire NHS Trust	RJN	57,654	54,709	106,484	4.45	74	-0.71	96
Royal Bolton Hospital NHS Foundation Trust	RMC	96,304	97,596	190,019	-2.22	107	-0.74	97
Basildon and Thurrock University Hospitals NHS Foundation Trust	RDD	126,107	123,547	241,501	0.74	96	-1.14	98
James Paget University Hospitals NHS Foundation Trust	RGP	89,874	85,015	166,199	4.32	76	-1.15	99
Portsmouth Hospitals NHS Trust	RHU	202,590	200,216	391,540	-0.18	100	-1.18	100
Homerton University Hospital NHS Foundation Trust	RQX	69,059	73,787	145,039	-8.14	131	-1.68	101
Stockport NHS Foundation Trust	RWJ	110,522	108,696	214,284	-0.50	101	-1.97	102
Mid Yorkshire Hospitals NHS Trust	RXF	190,394	190,544	375,639	-2.22	106	-1.97	103
Royal Liverpool and Broadgreen University Hospitals NHS Trust	RQ6	174,228	183,394	361,844	-7.11	124	-2.05	104
Medway NHS Foundation Trust	RPA	99,737	100,352	198,008	-2.82	111	-2.06	105
North West London Hospitals NHS Trust	RV8	142,050	147,003	290,579	-5.69	119	-2.23	106
United Lincolnshire Hospitals NHS Trust	RWD	203,175	193,264	382,147	2.57	86	-2.27	107
Oxford Radcliffe Hospitals NHS Trust	RTH	268,116	274,805	543,596	-4.85	116	-2.30	108
Burton Hospitals NHS Foundation Trust	RJF	72,679	70,793	140,121	0.07	97	-2.36	109
South Tees Hospitals NHS Trust	RTR	216,143	218,658	435,122	-4.17	115	-2.89	110
The Whittington Hospital NHS Trust	RKE	66,522	70,741	140,777	-8.84	136	-2.89	111
Nuffield Orthopaedic Centre NHS Trust	RBF	28,413	29,028	57,857	-5.26	117	-3.04	112
Airedale NHS Trust	RCF	61,364	58,321	116,311	1.78	90	-3.10	113
Yeovil District Hospital NHS Foundation Trust	RA4	55,643	52,233	104,250	2.97	81	-3.17	114
Scarborough and North East Yorkshire Health Care NHS Trust	RCC	56,909	53,247	107,086	2.53	87	-3.91	115
Derby Hospitals NHS Foundation Trust	RTG	204,701	200,934	404,552	-2.38	109	-4.01	116
Harrogate and District NHS Foundation Trust	RCD	65,928	61,913	125,292	1.51	92	-4.50	117
Weston Area Health NHS Trust	RA3	43,614	40,092	81,484	3.26	79	-4.92	118
Royal Free Hampstead NHS Trust	RAL	190,578	199,369	406,088	-9.46	138	-5.12	119
Royal Devon and Exeter NHS Foundation Trust	RH8	166,507	161,791	331,495	-3.10	112	-5.68	120
Sherwood Forest Hospitals NHS Foundation Trust	RK5	111,624	111,178	228,171	-5.62	118	-5.84	121
Mid Staffordshire NHS Foundation Trust	RJD	77,393	75,503	155,274	-3.84	113	-6.03	122
Nottingham University Hospitals NHS Trust	RX1	306,768	313,594	645,190	-8.27	132	-6.07	123
Maidstone and Tunbridge Wells NHS Trust	RWF	135,411	132,173	272,024	-3.97	114	-6.10	124
Sheffield Teaching Hospitals NHS Foundation Trust	RHQ	373,753	380,970	784,579	-8.10	130	-6.16	125
The Walton Centre for Neurology and Neurosurgery NHS Trust	RET	30,636	31,095	64,137	-7.85	129	-6.31	126
The Newcastle Upon Tyne Hospitals NHS Foundation Trust	RTD	328,446	342,872	711,606	-10.96	140	-6.89	127
South Devon Healthcare NHS Foundation Trust	RA9	104,861	99,222	207,178	-2.35	108	-7.45	128
South Tyneside NHS Foundation Trust	RE9	48,745	48,335	101,806	-7.63	128	-8.25	129
East and North Hertfordshire NHS Trust	RWH	140,316	131,414	276,827	-2.21	105	-8.26	130
Brighton and Sussex University Hospitals NHS Trust	RXH	171,517	168,943	358,048	-7.58	127	-8.81	131
Aintree University Hospitals NHS Foundation Trust	REM	120,660	120,798	258,061	-9.80	139	-9.54	132
Northern Lincolnshire and Goole Hospitals NHS Foundation Trust	RJL	140,019	137,524	294,771	-8.36	134	-9.84	133
Taunton and Somerset NHS Foundation Trust	RBA	110,791	106,734	228,841	-6.60	122	-9.86	134
University Hospitals Bristol NHS Foundation Trust	RA7	196,564	210,827	452,124	-16.13	144	-9.89	135

2009/10 Hospital Trust*	Provider code	Cost adjusted total outputs (Inpatient and Outpatient)	Quality adjusted total outputs (Inpatient) & Cost adjusted total outputs (outpatient)	Total NHS inputs (expenditure)	TFP cost-adj		TFP quality-adj	
					Standardised score	Ranking	Standardised score	Ranking
Northumbria Healthcare NHS Foundation Trust	RTF	146,052	141,338	303,631	-7.20	126	-10.04	136
Alder Hey Children's NHS Foundation Trust	RBS	64,456	75,320	162,258	-23.36	151	-10.29	137
Papworth Hospital NHS Foundation Trust	RGM	53,140	52,173	112,854	-9.16	137	-10.66	138
Wirral University Teaching Hospital NHS Foundation Trust	RBL	128,805	125,011	271,116	-8.34	133	-10.89	139
Blackpool, Fylde and Wyre Hospitals NHS Foundation Trust	RXL	132,909	125,588	272,496	-5.90	120	-10.93	140
East Kent Hospitals University NHS Foundation Trust	RVV	217,731	208,036	451,578	-6.98	123	-10.97	141
Salford Royal NHS Foundation Trust	RM3	138,431	147,952	322,000	-17.06	146	-11.20	142
Leeds Teaching Hospitals NHS Trust	RR8	360,643	379,813	826,770	-15.85	143	-11.22	143
Trafford Healthcare NHS Trust	RM4	41,644	41,048	91,555	-12.25	142	-13.36	144
York Hospitals NHS Foundation Trust	RCB	109,888	106,085	240,177	-11.73	141	-14.64	145
Imperial College Healthcare NHS Trust	RYJ	289,700	307,547	698,322	-19.97	150	-14.89	146
Northern Devon Healthcare NHS Trust	RBZ	59,383	55,139	125,440	-8.67	135	-15.05	147
King's College Hospital NHS Foundation Trust	RJZ	198,971	209,623	479,461	-19.94	149	-15.51	148
Central Manchester University Hospitals NHS Foundation Trust	RW3	242,717	271,297	624,354	-25.00	154	-16.03	149
Cambridge University Hospitals NHS Foundation Trust	RGT	220,693	227,878	525,343	-18.96	148	-16.17	150
University Hospital of South Manchester NHS Foundation Trust	RM2	136,648	136,121	317,106	-16.87	145	-17.04	151
North Tees and Hartlepool NHS Foundation Trust	RVW	110,423	111,942	261,337	-18.48	147	-17.22	152
Sheffield Children's NHS Foundation Trust	RCU	41,863	48,983	115,192	-29.89	160	-17.82	153
Barts and The London NHS Trust	RNJ	206,160	228,740	553,170	-28.10	157	-20.09	154
Birmingham Children's Hospital NHS Foundation Trust	RQ3	66,108	77,739	188,436	-32.32	161	-20.27	155
Royal Brompton and Harefield NHS Trust	RT3	75,284	80,766	201,421	-27.89	155	-22.51	156
Guy's and St Thomas' NHS Foundation Trust	RJ1	273,672	292,320	732,765	-27.95	156	-22.91	157
University College London Hospitals NHS Foundation Trust	RRV	197,835	213,945	538,221	-29.09	159	-23.18	158
Birmingham Women's NHS Foundation Trust	RLU	33,306	33,794	85,444	-24.80	153	-23.57	159
University Hospitals Birmingham NHS Foundation Trust	RRK	177,955	190,758	483,912	-29.05	158	-23.82	160
Great Ormond Street Hospital For Children NHS Trust	RP4	74,906	94,538	239,924	-39.77	163	-23.85	161
Gateshead Health NHS Foundation Trust	RR7	72,219	71,745	182,142	-23.51	152	-23.88	162
The Royal Marsden NHS Foundation Trust	RPY	60,081	66,683	188,288	-38.44	162	-31.56	163
Royal National Hospital for Rheumatic Diseases NHS Foundation Trust	RBB	5,425	5,641	17,938	-41.65	164	-39.22	164
The Christie NHS Foundation Trust	RBV	33,269	39,104	155,611	-58.75	165	-51.44	165
Clatterbridge Centre for Oncology NHS Foundation Trust	REN	12,323	13,603	60,561	-60.74	166	-56.59	166

* Hospital Trusts have been ordered according to the TFP quality-adj measure
